# Is the advanced age a contraindication to GERD laparoscopic surgery? Results of a long term follow-up

**DOI:** 10.1186/1471-2482-13-S2-S13

**Published:** 2013-10-08

**Authors:** Landino Fei, Gianluca Rossetti, Francesco Moccia, Teresa Marra, Paolo Guadagno, Ludovico Docimo, Marco Cimmino, Vincenzo Napolitano, Giovanni Docimo, Domenico Napoletano, Ludovica Guerriero, Beniamino Pascotto

**Affiliations:** 1 Division of Gastrointestinal Surgery Department of Anaesthesiological, Surgical and Emergency Sciences Second University of Naples-School of Medicine Via Pansini 5, 80131 Naples (Italy

## Abstract

**Background:**

In this prospective non randomized observational cohort study we have evaluated the influence of age on outcome of laparoscopic total fundoplication for GERD.

**Methods:**

Six hundred and twenty consecutive patients underwent total laparoscopic fundoplication for GERD. Five hundred and twenty-four patients were younger than 65 years (YG), and 96 patients were 65 years or older (EG). The following parameters were considered in the preoperative and postoperative evaluation: presence, duration, and severity of GERD symptoms, presence of a hiatal hernia, manometric and 24 hour pH-monitoring data, duration of operation, incidence of complications and length of hospital stay.

**Results:**

Elderly patients more often had atypical symptoms of GERD and at manometric evaluation had a higher rate of impaired esophageal peristalsis in comparison with younger patients. The duration of the operation was similar between the two groups. The incidence of intraoperative and postoperative complications was low and the difference was not statistically significant between the two groups. An excellent outcome was observed in 93.0% of young patients and in 88.9% of elderly patients (p = NS).

**Conclusions:**

Laparoscopic antireflux surgery is a safe and effective treatment for GERD even in elderly patients, warranting low morbidity and mortality rates and a significant improvement of symptoms comparable to younger patients.

## Background

Digestive diseases represent very common causes of morbidity and mortality in the elderly patients [1]. Among them gastroesophageal reflux disease (GERD) is usually more severe than in younger patients, and is frequently under-diagnosed and less treated [1]. The advent of laparoscopic surgery and its diffusion [2], has also greatly reduced the morbidity of fundoplication antireflux surgery and now it is considered the surgical treatment of choice for GERD [3,4]. The aim of our study is to compare the outcome of young and elderly patients undergoing laparoscopic antireflux surgery for the treatment of GERD.

## Materials and methods

From September 1992 to December 2011, 620 consecutive patients, 269 male and 351 female, mean age 43.7 years (range 12-81) with GERD underwent laparoscopic Nissen-Rossetti fundoplication. The preoperative and postoperative data were prospectively collected. Demographic data were obtained at the time of first visit. Ninety-six patients older than 65 years of age (40 M, 56 F) (mean age 73.1 ± 3.4) were defined as the elderly group (EG) whereas the remaining 524 younger than 65 years of age (229 M, 295 F) (mean age 47.8 ± 2.9) were defined as the young group (YG). Ethics board approval for collecting and using these data was obtained.

## Preoperative study

Preoperatively all patients underwent clinical and instrumental examinations. The medical evaluation included a structured questionnaire based on modified DeMeester symptom scoring system **(**Table 1**)**. Instrumental examinations comprised upper gastrointestinal endoscopy, esophageal manometry and 24-h pH monitoring; for pHmetric and manometric examinations, patients had to suspend at least for a week the assumption of antisecretive and antiacid drugs, and stop the assumption of prokinetic drugs and other pharmaceutical products that could interfere with the basal tone of the lower esophageal sphincter (LES) (nitrates, calcium-antagonists, aminophylline, beta2-agonists, etc) at least 24 hours before the investigations [5-7]. Esophagitis severity was assessed by means of Savary-Miller grading system. Patients with esophageal strictures and/or paraesophageal hernias were excluded from the study.

**Table 1:** Modified DeMeester scoring system

*Symptoms*	*Score*	*Description*
Dysphagia	0 1 2 3	None Occasional transient episodes Require liquids to clear Impactionrequiring medical attention
Heartburn	0 1 2 3	None Occasional brief episodes Frequent episodes requiring medical treatment Interference with daily activities
Regurgitation	0 1 2 3	None Occasional episodes Predictable by posture Interference with daily activities

**• Esophageal manometry: **Different parameters were evaluated: (1) LES pressure; (2) LES relaxation in response to swallowing; (3) amplitude and propagation of peristalsis (esophageal peristalsis was considered impaired when <30 mmHg). The LES was studied by both the stationary and the rapid pull-through methods.

**• 24 hours pH-monitoring: **The electrodes were placed, respectively, 5 cm above the proximal margin and 5 cm below the distal margin of the LES, identified by means of stationary manometry [8,9].

## Postoperative study

On an outpatient basis, the patients came to our department each six months for the first postoperative year and, after, each year and were invited to fulfill a standardized questionnaire dealing with presence of typical or atypical symptoms and based on the modified DeMeester score **(**Table 1**)**. Satisfaction of the procedure and the will of undergoing the same operation after knowing its effects were defined as excellent outcome.

Instrumental follow-up included: esophageal manometry (performed at 6 months, 1 year, and 2 years after surgery) and 24h pH monitoring (performed at 1 year after surgery).

## Statistical analysis

It was carried out using SPSS for Windows (version 12.0, SPSS Inc. Chicago, IL). Results were expressed as mean ± SD unless otherwise indicated. Student's *t-*test, the Chi-square test, the Fischer's exact test and the Wilcoxon signed rank test were used as appropriate. Correlations among the various parameters were analysed using Fischer's exact test. The Wilcoxon signed rank test was used to compare the preoperative and postoperative modified DeMeester symptom score. *P *value < 0.05 was considered statistically significant.

## Results

In the YG, the mean duration of preoperative symptoms was 4.2 ± 2.1 years (range 1-12) whereas in the EG it was 8.5 ± 2.7 years (range 4-21). Incidence and severity of preoperative symptoms in the two groups are summarized in Table 2 and Table 3. At manometric evaluation, no statistically significant differences in the mean LES pressure were found when the two groups were compared (p = NS) but the EG had a higher rate of impaired esophageal peristalsis (defined as peristaltic waves with a pressure value lower than 30 mmHg) in comparison with their younger counterparts (*P *< 0.05) **(**Table 4**)**. Incidence of Hiatal Hernia (HH) was 88.5% (85/96) in elderly patients and 71.7% (376/524) in young patients (P < 0.05). Table 5 shows the prevalence of HH and esophagitis and pH metric values either in elderly and younger patients. In the EG, 69.8% of the patients(67/96) presented esophagitis: 16 of 67 (23.9%) had a grade I esophagitis while 51 out of 67 (76.1%) had a grade II-III esophagitis. In the YG, 34.9% of the patients (183/524) presented esophagitis: 111 out of 183 (60.6 %) had a grade I esophagitis while 72 of 183 (39.3%) had a grade II-III esophagitis. Therefore, in the EG, a significant higher grade of esophagitis has been found along with a higher incidence of Barrett esophagus **(**Table 5**)**. A pathologic DeMeester score was found at pH-monitoring in all patients of both subgroups: in the YG, it was 12.6 ± 1.3 whereas in the EG it was 13.2 ± 1.3 (p = NS). The mean percentage of total time at pH< 4 observed at 24-h pH monitoring in both groups is shown in Table 5: there was a significant major incidence of acid reflux in EG, either total either in supine and upright position.

**Table 2:** Incidence of preoperative symptoms in EG and YG

*Symptoms*	*EG (%)*	*YG (%)*	*P*
Heartburn	67/96(69.8%)	475/524(90.6%)	<0.05
Acid regurgitation	58/96(60.4%)	461/524(88.0%)	<0.05
Solid food dysphagia	37/96(38.5%)	41/524(7.8%)	<0.05
Chest pain	25/96(26.0%)	70/524(13.4%)	<0.05
Respiratory complication (chronic cough,sleep apnea,asthma,laryngitis)	40/96(41.7%)	24/524(4.6%)	<0.05

**Table 3:** Severity of preoperative symptoms in EG and YG

Symptoms	EG	YG	*P*
Heartburn	1.6 ± 0.89	2.8 ± 0.76	<0.05

Acid regurgitation	1.6 ± 0.98	2.4 ± 0.91	<0.05

Solid food dysphagia	1.7 ± 0.78	0.6 ± 0.22	<0.05

Chest pain	1.7 ± 0.84	1.5 ± 0.89	>0.05

Respiratory complication	1.9 ± 1.06	1.1 ± 0.47	<0.05

**Table 4:** Preoperative manometric evaluation in EG and YG

*Manometry*	*EG*	*YG*	*P*
LES pressure (mmHg)	10.8 ± 1.6	11.1 ± 1.4	>0.05
Impaired esophageal peristaltis (<30 mmHg)	64/96 (66.6%)	179/524 (34.2%)	<0.05

**Table 5:** Preoperative evaluation: incidence of hiatal hernia, esophagitis, Barrett and pHmetric data in EG and YG

	EG	YG	P
Hiatal Hernia	85/96 (88.5%)	376/524 (71.7%)	<0.05

Esophagitis	67/96 (69.8%)	183/524 (34.9%)	<0.05

Barrett	7/96 (7.3%)	14/524 (2.7%)	<0.05

De Meester score	13.2 ± 1.3	12.6 ± 1.3	>0.05

(%)time pH<4 (total)	12 ± 2	7 ± 2	<0.05

(%)time pH<4 (supine)	13 ± 3	8 ± 2	<0.05

(%)time pH<4 (upright)	10 ± 4	5 ± 3	<0.05

All the interventions were completed via laparoscopic approach. Mean operative time was 48 ± 13 min in YG and 51 ± 15 min in EG (p = NS). Intraoperative blood loss was similar (30 ± 10 ml vs 35 ± 15 ml, respectively in YG and EG) (p = NS). No mortality was observed in both groups. A major complication occurred in 4/524 patients (0.7%), all among the YG (p = NS). Mean postoperative hospital stay was 2.5 ± 0.8 days in YG patients (range 1-5) and 2.7 ± 1.1 days in EG patients (range 1-6) (p:NS). Normal activity resumed in 8.4 ± 3.5 days in YG and 11.6 ± 8.7 days in EG (p < 0.05).

We followed up clinically 589 (95.0%) of 620 patients, 90 (93.8%) patients in the EG and 499 (95.2%) patients in YG. Two patients in the EG died four years after surgery for no surgery correlated event. In the YG, the mean follow-up was 89.7 ± 8 months (range 6-180) whereas in EG it was 71.2 ± 9 months (range 6-107). An excellent outcome was observed in 464/499 (93.0%) younger patients and in 80/90 (88.9%) elderly patients (p = NS). Both groups showed significant improvement in clinical symptom score **(**Table 6**)**. At 6 months, persisting postoperative dysphagia (DeMeester score 2-3) leading to >15% of weight loss was observed in 16 of 499 patients (3.2%) in YG **(**Table 7**)**. In EG, persisting postoperative dysphagia was observed in 3 of 90 patients (3.3%) (p = NS) **(**Table 7**)**. No statistically significant difference was observed between patients with normal and impaired peristalsis. Seven patients in YG and the three patients in EG were treated with endoscopic dilatation, whereas the remaining nine patients in YG underwent a laparoscopic redo-funduplication with partial resolution of dysphagia. Recurrent heartburn was observed and confirmed by means of esophageal 24 h pH monitoring in 20/589 patients (3.4%), which was due to a disrupted wrap, an herniated wrap, and a slipped Nissen detected at X-ray barium in nine, six, and five cases, respectively. Fifteen patients resumed their antisecretory drugs; the remaining five patients, all in YG, underwent redo-fundoplication with partial resolution of symptoms. Respiratory symptoms showed a significant improvement in both groups. Other data regarding hyper-flatulence, early satiety and bloating are showed in Table 7. Esophageal manometric follow-up (performed at 6, 12, and 24 months after surgery) was made in 483 of 589 patients (82.0%) at 6 months (69/90, (76.7%) in EG and 414/499, (83.0%) in YG), 403/589 (68.4%) at 12 months (55/90, (61.1%) in EG and 348/499, (69.7%) in YG), and 391/589 (66.4%) at 24 months (52/90, (57.8%) in EG and 339/499, (67.9%) in YG). Stationary esophageal manometry showed a significant improvement in the mean new high pressure zone (N-HPZ) value in comparison with preoperative values in the two groups (p < 0.05) **(**Table 8**and Figure 1)**. Manometric evaluation at 24 months after surgery showed an increase of mean peristalsis waves in 39/52 patients of the EG (75.0%) and in 149/339 patients of the YG(44.0%) **(**Table 8**)**. Twenty-four hour pH monitoring at 1 year after surgery was performed in 278/589 (47.2%) patients. There was a significant postoperative decrease in esophageal DeMeester score and percentage of time at pH<4 during 24 h in both groups **(**Table 9**)**.

**Table 6:** Pre/postoperative symptoms score in EG and YG (mean symptom score±SD)

*Symptoms*	*________EG_______*		*P*	*_______YG_______*		*P*
	***Preop*.**	***Postop*.**		***Preop*.**	***Postop*.**	
Heartburn	1.6 ± 0.89	0.3 ± 0.14	<0.05	2.8 ± 0.76 2.8 ± 0.76	0.4 ± 0.11	<0.05
Acid regurgitation	1.6 ± 0.98	0.4 ± 0.15	<0.05	2.4 ± 0.91	0.3 ± 0.14	<0.05
Solid food dysphagia	1.7 ± 0.78	0.5 ± 0.14	<0.05	0.6 ± 0.22	0.3 ± 0.17	<0.05
Chest pain	1.7 ± 0.84	0.4 ± 0.24	<0.05	1.5 ± 0.89	0.3 ± 0.15	<0.05
Respiratory complication	1.9 ± 1.06	0.4 ± 0.13	<0.05	1.1 ± 0.47	0.3 ± 0.14	<0.05

**Table 7:** Postoperative side effects in EG and YG

	*EG*	*YG*	*P*
***Side effects:*** *number patients (%)*			
Dysphagia	3/90 (3.3%)	16/499 (3.2%)	>0.05
Heartburn	3/90 (3.3%)	17/499 (3.4%)	>0.05
Hyperflautolence	2/90 (2.2%)	8/499 (1.6%)	>0.05
Early satiety	2/90 (2.2%)	11/499 (2.2%)	>0.05
Bloating	3/90 (3.3%)	6/499 (3.2%)	>0.05
Chest pain	1/90 (0.1%)	2/499 (0.4%)	>0.05

**Table 8:** Postoperative manometric evaluation at 24 months after surgery in EG and YG

***Manometry***	***________EG_______***		***P***	***_______YG_______***		***P***
	***Preop*.**	***Postop*.**		***Preop*.**	***Postop*.**	
N-HPZ pressure (mmHg)	10.8 ± 1.6	28.3 ± 1.6	<0.05	11.1 ± 1.4 2.8 ± 0.76	28.2 ± 1.3	<0.05
Increase of mean peristalsis	39/52(75.0%)			149/339(44.0%)		
waves patients n(%)						

**Table 9:** Preoperative and postoperative DeMeester score and percentage of esophageal reflux time during 24 h in EG and YG

	*Preoperative*	*1 yr after surgery*
**DeMeester score** EG YG	13.2 ± 1.3 12.6 ± 1.3	1.3 ± 0.7 1.2 ± 0.2
**(%) time pH<4** EG YG	12 ± 2.0 7.0 ± 2.0	1.5 ± 0.3 0.9 ± 0.8

## Discussion

Gastroesophageal reflux disease (GERD) is one of the most common disorders of the upper gastrointestinal tract. Symptoms occur in approximately 20% of adults among western population [10] with great impact on life quality and elevated social costs [1]. GERD ethiopatogenesis is certainly multifactorial with alterations that include a less effective antireflux barrier, defective esophageal-clearance, altered esophageal mucosal resistance, and delayed gastric emptying [11]. Hiatal hernia as a structural defect of the antireflux barrier is a determining factor of GERD, by impairing both the diaphragmatic component and the clearance of acid refluxate from the distal esophagus [12-17]. GERD has nearly the same prevalence among elderly and young people, but older patients have more severe esophageal mucosal injuries like grade 3 or 4 esophagitis and Barrett disease [18-23]. Furthermore, also hiatal hernia incidence seems greater in elderly patients (about 60% in patients >60 years old) [24].

Also in our study, the elderly group (EG) had a higher rate of erosive esophagitis (69.8% vs 34.9.%) and a lower rate of Grade I esophagitis (23.9% vs 60.6%). Moreover, incidence of Barrett esophagus as well as mean percentage of total time < 4 at pH-monitoring were significantly higher in the EG. Furthermore, we noted a significant higher rate of hiatal hernia in the EG (88.5% vs 71.7%) **(**Table 5**)**.

Previous studies observed esophagitis and Barrett Esophagus in 81% of patients aged 60 years vs 47% of young people (p < 0.002) [20]. Zhu [19] and Pizza [25] found that the percentage of time with pH<4 was greater in older patients with GERD than in younger ones (p < 0.05). Furthermore, among elderly patients with esophagitis, nearly 21% had grade III-IV disease compared with only 3.4% of younger patients (p < 0.002) [19]. Cameron [18] demonstrated that the prevalence of Barrett esophagus increased with age to reach a plateau by the seventh decade. Fass [22] reported that the mean incidence rate of erosive esophagitis was 74% in the elderly and 64% in the younger patients and the frequency of symptoms was lower in the elderly group.

Many factors associated with ageing could be implicated in the different severity of mucosal injuries, such as increased gastric acid secretion, reduced salivary bicarbonate secretion, delayed esophageal and gastric emptying, disordered esophageal peristalsis, increased incidence of hiatal hernia and frequent use of injurious medications for esophageal mucosa (like non steroidal anti-inflamatory drugs) [26]. We found elderly often present with less severe heartburn and acid regurgitation (Tables 2, 2). It is not clear which factors reduce frequency and severity of these symptoms: altered esophageal pain perception to acid in the elderly may be the result of an ageing process [22]. On the other hand, we observed higher frequency and severity of atypical symptoms on elderly GERD-affected patients, such as chest pain, respiratory complications (chronic cough, sleep apnea, asthma, laryngitis) and dysphagia for solid foods (Tables 2, 3). These characteristics surely make prevalence of GERD underestimated in elderly population.

Despite this, in Western Countries life expectancy increased and keep on increasing [23], so always more GERD-affected patients are 65 years old or more. This rapidly enlarges demands for effective therapy with greater costs for health care systems all over the world. According to this, it is necessary to evaluate both effectiveness and safety of laparoscopic fundoplication in patients more than 65 years old in comparison with this surgical approach in younger patients.

Surgical correction of GERD has been shown to be a cost-effective treatment by reducing long-term

complications such as Barrett esophagus and stricture and by eliminating the need of a life-long medical therapy especially for young patients [27-36]. However, a high morbidity and mortality rate of open surgery performed in the elderly, limited the number of these patients referred to surgery [37]. Instead, in elderly population with GERD, laparoscopic surgery has proven to be effective with low morbidity and mortality rates: Richter [1] and Pizza [25], observed that laparoscopic Nissen fundoplication did not increase the mortality, morbility and hospital stay in the elderly patients compared to younger surgical patients. Kamolz [38] showed that age should not be considered a contraindication to laparoscopic surgical treatment of GERD as 97% of elderly patients would choose surgical treatment again if necessary. Also in our study we confirmed these excellent results: except for preoperative disease severity, we did not find any significant difference in perioperative and postoperative results as well as in subjective and objective outcome between the two groups.

After surgical treatment we found significant improvement in heartburn, acid regurgitation, chest pain and respiratory complications of GERD in both EG and YG **(**Table 6**)**. An excellent outcome was observed in 464/499 (93.0%) younger patients and in 80/90 (88.9%) elderly patients. Persisting dysphagia occurred in 16/499 (3.2%) in YG and 3/90 (3.3%) in EG while 17/499 (3.4%) in YG and 3/90 (3.3%) in EG had recurrence of heartburn (p = NS). Differences between the two groups were not statistically significant also regarding the incidence of other side effects (flatulence, early satiety, etc) **(**Table 7**)**.

There have been debates in literature regarding the realization of partial fundoplication in patients with defective esophageal peristalsis, and it seemed reasonable therefore, to choose this kind of wrap in elderly patients. Many Authors supported the realization of a partial fundoplication in patients with impaired esophageal peristalsis to lower the incidence of persistent postoperative dysphagia [39-41] because partial wrap was considered as effective as total wrap to control gastroesophageal reflux, and short-term follow-up seemed to validate the choice of partial fundoplication [42,43]. Later on, partial antireflux procedure showed its inadequacy to assure a good protection from reflux at a long-term follow-up [44-46]. Livingston [47] reported a 1.4% recurrence rate of reflux in patients with total fundoplication versus 6.7% in those with partial fundoplication. At a long-term follow-up, Fernando [48] observed that 38% of Toupet patients used PPI versus 20% with Nissen. Jobe [49], in a ten years follow-up, noted a recurrence rate for reflux until 51% in patients treated with partial fundoplication (Toupet and Dor). Moreover, total fundoplication seems not to determine a higher incidence of postoperative dysphagia compared with the partial wraps, even in patients with impaired peristalsis [50-53]. In a prospective randomized trial, Bessell [54] concluded that calibrating the antireflux wrap according to esophageal motility was not necessary, because the postoperative persistent dysphagia rate was similar between patients with total or partial wrap. Velanovich [55] did not find any statistically significant difference in postoperative dysphagia rate related to esophageal motility disorders (MD) (15.8% MD+ versus 16.4% MD-) in a group of patients undergoing total fundoplication.

Besides, total wrap seems to bring about an improvement of esophageal peristalsis. Heider [56] observed an increase of 47% of mean peristaltic waves in distal esophagus compared with preoperative time (p < 0.01), with the normalization of the esophageal motility in 74% of patients. Oleynikov [57] in a trial comparing total and partial fundoplication noticed that in patients undergoing partial wrap, the mean amplitude of peristaltic waves increased from 27.8 mmHg before surgery to 35.6 mmHg postoperatively (p < 0.05), while in patients treated with total fundoplication, these values were respectively 28.2 mmHg versus 49.0 mmHg (p < 0.05). These evidences strongly support the choice of performing a total fundoplication even in elderly patients. Also our choice, since 1972, has always been favorable to the total Nissen-Rossetti fundoplication [29,30,50]. Routinely, we perform intraoperative endoscopy and manometry in order to calibrate antireflux wrap [29,30,50]. Usually, we calibrate the N-HPZ at values ranging from 20 to 45 mmHg ('hypercalibrated Nissen'), building the wrap around the gastroscope (with a diameter of 9 mm). This hypercalibration, in contrast with the 'floppy Nissen' of Donahue and DeMeester [58], resulted from the retrospective evaluation of a former series in which we used to calibrate the fundoplication to pressure values similar to those of a normal sphincter ('normocalibrated Nissen': 10-20 mmHg). This experience was followed by a high rate of gastroesophageal reflux recurrence (28.5%) in the first 12 months after surgery [59], demonstrating that high pressure zone (HPZ) values of the Nissen-Rossetti wrap decrease after surgery with time. Our preference for total calibrated wrap led us to consider it also in the treatment of patients affected with severe motility disorders such as achalasia and epiphrenic diverticula with excellent outcome [50,60-62]. Also evidences from this study confirm our choice, as excellent results have been observed either in elderly either in young group, with no differences in function of esophageal peristalsis impairement.

## Conclusions

In conclusion, even in elderly patients, laparoscopic antireflux surgery is a safe and effective treatment for GERD, with low morbidity rates and improvement of symptoms comparable to younger patients. Total Nissen-Rossetti fundoplication represents the best therapeutic choice even in this group of patients.

## Competing interests

The Authors declare that there is no conflict of interest with any financial organization regarding the material discussed in the manuscript.

## Authors' contributions

L.F.: Concept of the study and drafting of the manuscript

G.R.: Concept of the study and drafting of the manuscript

F.M.: Drafting and correction of the manuscript

T.M.: Instrumental preoperative and postoperative evaluation

P.G.: Clinical preoperative and postoperative evaluation

L.D.: Clinical preoperative and postoperative evaluation

M.C.: Instrumental preoperative and postoperative evaluation

V.N.: Drafting and correction of the manuscript

G.D.: Statistical analysis

D.N.: Instrumental preoperative and postoperative evaluation

L.G.: Clinical preoperative and postoperative evaluation

B.P.: Statistical analysis

## Authors' information

L.F.: Full Professor of Surgery at Second University of Naples, Chief Division of Gastrointestinal Surgery at Second University of Naples.

G.R.: Resident in Surgery at Second University of Naples

F.M.: Resident in Surgery at Second University of Naples

T.M.: Resident in Surgery at Second University of Naples

P.G.: Associate Professor of Surgery at Second University of Naples

L.D.: Full Professor of Surgery at Second University of Naples

M.C.: Resident in Surgery at Second University of Naples

V.N.: Assistant Professor of Surgery at Second University of Naples

G.D.: Associate Professor of Surgery at Second University of Naples

D.N.: Resident in Surgery at Second University of Naples

L.G.: Resident in Surgery at Second University of Naples

B.P.: Resident in Surgery at Second University of Naples

## Declarations

Funding for this article came from personal funds.

This article has been published as part of *BMC Surgery *Volume 13 Supplement 2, 2013: Proceedings from the 26th National Congress of the Italian Society of Geriatric Surgery. The full contents of the supplement are available online at http://www.biomedcentral.com/bmcsurg/supplements/13/S2
